# Distinct pathways for zinc metabolism in the terrestrial slug *Arion vulgaris*

**DOI:** 10.1038/s41598-019-56577-7

**Published:** 2019-12-27

**Authors:** Martin Dvorak, Raimund Schnegg, Willy Salvenmoser, Òscar Palacios, Herbert Lindner, Oliver Zerbe, Armin Hansel, Markus Leiminger, Gerhard Steiner, Reinhard Dallinger, Reinhard Lackner

**Affiliations:** 1 Institute of Zoology and Center of Molecular Biosciences, University of Innsbruck, Technikerstrasse 25, A-6020 Innsbruck, Austria; 2 Departament de Química, Facultat de Ciències, Universitat Autònoma de Barcelona, E-08193 Cerdanyola del Vallès, Barcelona, Spain; 3 Institute of Clinical Biochemistry, Biocenter, Innsbruck Medical University, Innrain 80, A-6020 Innsbruck, Austria; 4 Department of Chemistry, University of Zurich, 8057 Zurich, Switzerland; 5 Institute for Ion Physics and Applied Physics, University of Innsbruck, Technikerstrasse 25, A-6020 Innsbruck, Austria; 6 GRIMM Aerosol Technik Ainring GmbH & Co. KG, 83404 Ainring, Germany

**Keywords:** Metals, Mass spectrometry, Metabolic pathways, Animal physiology

## Abstract

In most organisms, the concentration of free Zn^2+^ is controlled by metallothioneins (MTs). In contrast, no significant proportions of Zn^2+^ are bound to MTs in the slug, *Arion vulgaris*. Instead, this species possesses cytoplasmic low-molecular-weight Zn^2+^ (LMW Zn) binding compound that divert these metal ions into pathways uncoupled from MT metabolism. Zn^2+^ is accumulated in the midgut gland calcium cells of *Arion vulgaris*, where they associate with a low-molecular-weight ligand with an apparent molecular mass of ~ 2,000 Da. Mass spectrometry of the semi-purified LMW Zn binding compound combining an electrospray ion source with a differential mobility analyser coupled to a time-of-flight mass spectrometer revealed the presence of four Zn^2+^-containing ion signals, which arise from disintegration of one higher MW complex resulting in an ion-mobility diameter of 1.62 nm and a molecular mass of 837 Da. We expect that the novel Zn^2+^ ion storage pathway may be shared by many other gastropods, and particularly species that possess Cd-selective MT isoforms or variants with only very low affinity to Zn^2+^.

## Introduction

Zinc (Zn^2+^) is an essential metal ion and co-factor in the active centre of many enyzmes^1^. However, excess amounts of Zn^2+^ are toxic and can adversely interact with the metabolism of other essential metal ions including, for example, copper, iron, and others^2^. In particular, metallothioneins (MTs) can bind transition metal ions with high affinity through the sulphur atoms of their cysteine residues. They are known to play a crucial role in regulation, inactivation and metabolism of trace elements^3,4^. Most of our knowledge about these proteins comes from vertebrate MTs. The overall picture is that vertebrate MTs are considered to be largely unspecific and ready to scavenge in their two metal binding domains different metal ions like Cu^+^, Cd^2+^, Zn^2+^, and many others, simultaneously^5^.

The promiscuous metal binding property of vertebrate MTs has two major implications: Firstly, native MTs are likely to be saturated with the more abundant Zn^2+^ ions, releasing them when metal ions with a higher affinity like Cd^2+^ are encountered, serving to detoxify Cd^2+^ ions. Secondly due to the metal-unspecific nature of vertebrate MTs they can immobilize different metal ions simultaneously and therefore play a general role in metal ion homeostasis.

Since terrestrial gastropods needed to adapt to terrestrial life and cope with excessive water losses, they also face the problem of uptake and excretion of unwanted and toxic trace elements. Probably as an adaptation to this necessity, most terrestrial gastropod species evolved mechanisms to express metal-selective MTs^6,7^. The terrestrial slug *Arion vulgaris*, for example, expresses a highly Cd-selective MT (AvMT1) that prevalently handles the detoxification of this harmful metal ion in its midgut gland^4^.

As a consequence of this metal specificity, the metabolism of the essential trace element Zn^2+^ is no longer handled by the MT turntable. In fact, most Cd-selective MT isoforms of snails contain only traces of Zn^2+^ after native purification from midgut gland extractions^6^ (Dallinger *et al*. 2020, submitted). Such a metabolic separation of Zn^2+^ pathways is also suggested by the fact that upon gel chromatography of midgut gland extracts from *Arion vulgaris*, the Cd-containing fractions assigned to MT are clearly separated from the Zn-containing fractions which elute at a much lower molecular weight. This suggest that in invertebrates like *Arion vulgaris*, there may be specific pathways for different metal ions and indicate that the expression of cadmium selective MTs requires the presence of more specific pathways for other metal ions like Zn^2+^. Thus, when there is a metal-specific MT on one side there needs to be more specific pathway for other metals. To shed light on this apparently distinct handling of Zn^2+^ in this species, we address in the present study the Zn metabolism of *Arion vulgaris* by trying to give a short characterization of involved compounds and discuss the possible involvement of already known ligands and pathways in Zn^2+^ accumulation and sequestration. Our conclusion is that in animals with highly metal-selective MTs for discrimination of Cd^2+^ and/or Cu^+^ -specific pathways (like in terrestrial snails), even more specific mechanisms are needed for handling of other metals such as the essential trace element Zn^2+^.

## Results

**Figure 1:**
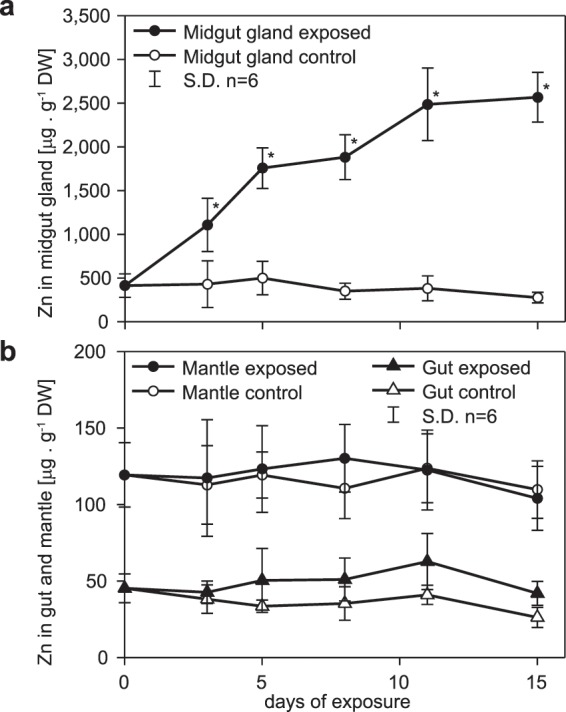
Zinc concentrations (means and standard deviations, n = 6) expressed in µg per g dry weight, shown in organs of slugs exposed to Zn over a period of 15 days. (**a**) Course of Zn concentrations through 15 days in midgut gland of control (open circles) and Zn-exposed slugs (full circles). (**b**) Zn concentrations through 15 days in gut (triangles) and mantle (circles) of control (open symbols) and Zn-exposed slugs (full symbols). The asterisks above single values indicate significant differences compared to respective control values (Holm–Sidak method of all pairwise multiple comparison) (p ≤ 0.05).

As previously shown for Cd and Cu^4^, also Zn is predominantly accumulated in the midgut gland of *Arion vulgaris* (Fig. 1). In exposed animals, Zn concentrations as high as 2,568.7 µg · g^−1^ dry weight were assessed in this organ, compared to concentrations of 277.0 µg · g^−1^ dry weight in control slugs. When fed with Zn-enriched lettuce (941.9 µg · g^−1^ dry weight), Zn concentrations in the midgut gland were increased by a factor of 2.7. In contrast, no significant variations of Zn concentrations were observed in the mantle and gut tissues between control and Zn-exposed slugs throughout the whole period of exposure (Fig. 1b).

The accumulation of Zn in the midgut gland was expected and confirms the central role of this organ in metal accumulation of gastropods^8–10^. However, the pathways of Zn metabolism in this organ remained still unresolved. To obtain a complete picture of Zn metabolism in *Arion vulgaris* as a model species, we investigated the cellular distribution of Zn^2+^ in different tissues, focusing on cellular localization, purification and characterization of involved compounds and pathways in the slug’s midgut gland, and compared them to known pathways.

### Histochemistry

**Figure 2:**
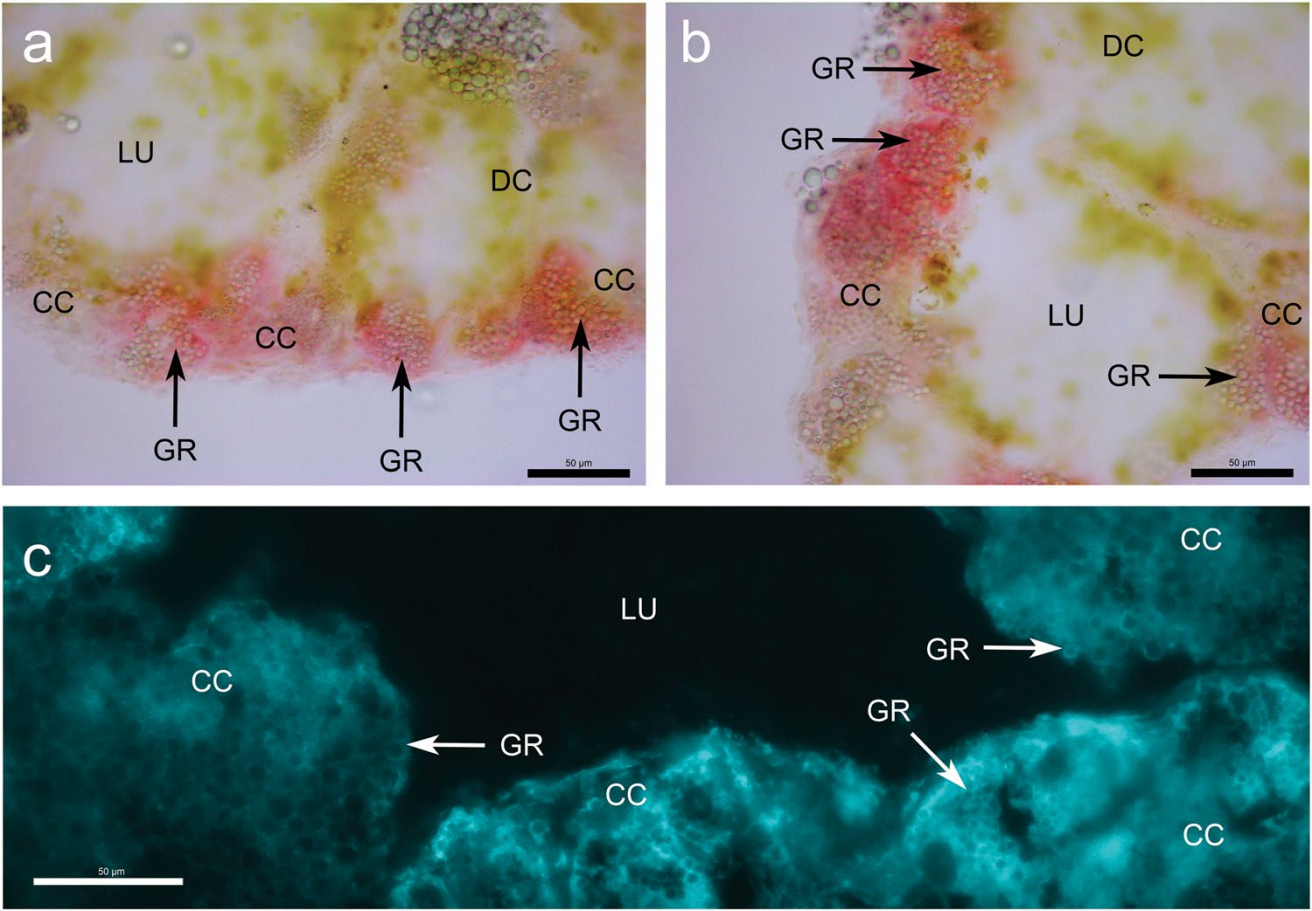
Zinc distribution in midgut gland sections of zinc-exposed *Arion vulgaris* with colour dithizone staining (**a**,**b**) and fluorescent toluenesulfonamidoquinoline (TSQ) staining (**c**). Midgut gland cross sections showing lumen (LU) surrounded by digestive cells (DC) and calcium cells (CC) containing calcium granules (GR). Zinc stained by dithizone (red colour) (**a**,**b**) is exclusively allocated in calcium cells. Fluorescent staining by TSQ localize zinc (bright blue colour) (**c**) mainly in cytoplasm of calcium cells and on the outer edge of calcium granules (**c** - GR). The bar corresponds to a size of 50 µm.

First, we applied histochemistry to gain information about the cellular localization and compartmentalization of Zn^2+^ in midgut gland cells of *Arion vulgaris*. Tissue Zn^2+^ ion distribution in midgut gland sections was visualized by either dithizone staining or toluenesulfonamidoquinoline (TSQ) fluorescence detection. Although dithizone is not absolutely specific for Zn^2+^, a strong signal was observed exclusively in the midgut gland calcium cells, especially visible in Zn-exposed animals (Fig. 2a,b). Localization of Zn^2+^ was confirmed by the strong Zn-specific fluorescence signal of TSQ (Ex/Em: 380/495), observed mainly in cytoplasm of calcium cells and on the outer edge of calcium granules (Fig. 2c). The fluorescent signal in control animals was much weaker. Nevertheless, calcium granules as well as cytoplasm were still stained, confirming the presence of basal amounts of Zn^2+^ ions in the controls, as expected, and thereby validating in this way the used methodology. Overall, histochemical images indicate that Zn^2+^ is particularly present in the cytoplasm of midgut gland calcium cells, indicating that the metal is largely associated there with one or a few specific ligands that can be visualised by histochemistry.

### Purification of the low molecular weight Zinc complex (LMW Zn)

Having observed a major proportion of Zn in the cytoplasmic compartment of calcium cells, the question arose about what the chemical nature of this metal pool might be. To this scope, we applied different chromatographic methods, considering that in previous studies on metal accumulation in gastropod midgut gland, Zn was found to be associated mainly with low-molecular weight fractions^11,12^ that remained uncharacterized.

**Figure 3:**
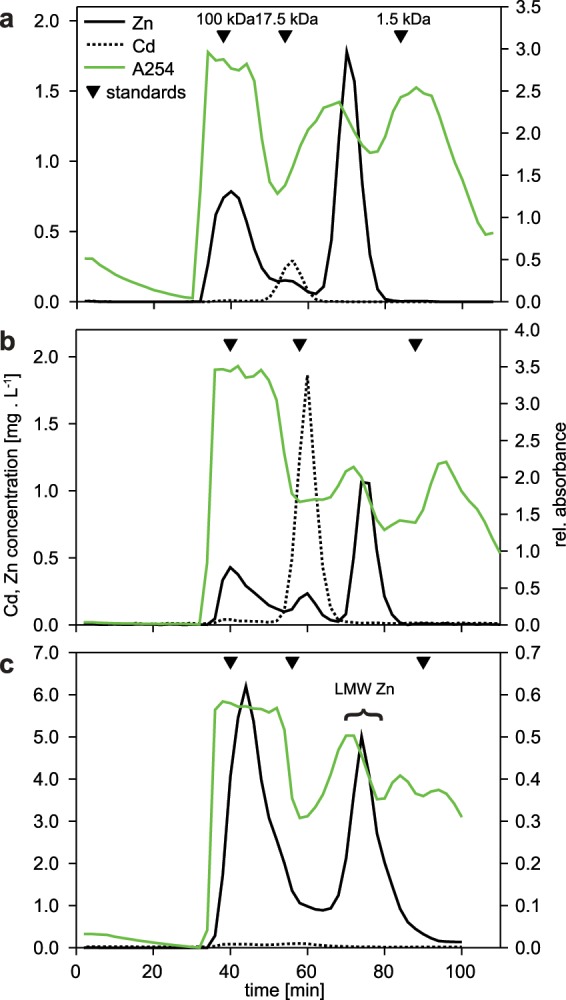
Gel permeation chromatography (Sephacryl S-100, 25 × 300 mm) profiles of midgut gland homogenate supernatants from control (**a**), Cd (**b**) and Zn-exposed *Arion vulgaris* (**c**), showing absorptions 254 nm (green line), as well as concentrations of Cd (dotted line) and Zn (black line), as specified in (**a**). Elution peaks of calibration standards (blue dextran, ≥100 kDa; myoglobin, 17.5 kDa; and vitamin B12, 1.4 kDa) are marked by inverted black triangles above the elution profiles. Fractions collected and pooled for subsequent purification (Fig. 4c) are indicated by brace and LMW Zn label.

First, midgut gland homogenates were fractionated by gel filtration chromatography into mainly two distinct fractions (Fig. 3). The high-molecular-weight fraction, which correlates with the void volume of the column, corresponded to an apparent molecular weight ≥100 kDa, while the interesting low-molecular-weight Zn fraction was eluted in the range between 17 and 1 kDa (Fig. 3). This was consistent for control (Fig. 3a), Cd (Fig. 3b) and Zn-exposed (Fig. 3c) animals. In Zn-exposed individuals, the metal was distributed almost equally between low and high-molecular weight fractions (Fig. 3c). We assume that the high-molecular weight Zn fraction represents Zn-containing proteins and enzymes of various biological functions^1^, whereas the low-molecular-weight Zn-containing fraction was suggested to represent the Zn pool visualised by histochemistry, containing one or more novel Zn-binding ligands (Fig. 3c). These ligands were also present in control slugs (Fig. 3a). In addition, we also observed very low amounts of Zn ions in a range of fractions around 17.5 kDa, especially in the Cd-exposed animals (Fig. 3a,b), likely corresponding to MTs^4^.

**Figure 4:**
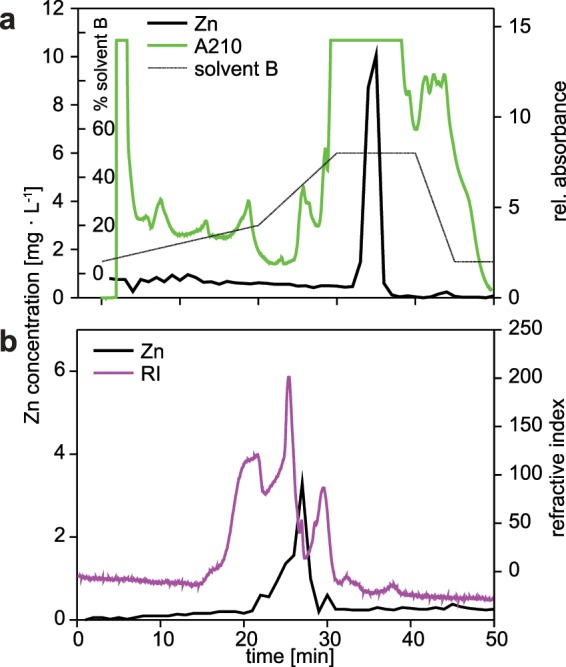
HPLC separation profile with (**a**) NH_2_-column of pooled zinc-containing low molecular weight fractions of gel chromatography showing absorptions at 254 nm (green line), Zn concentration (thick black line) and solvent gradient (thin black line), (**b**) with Superdex peptide 10/300 GL column of pooled zinc-containing fractions after amino column separation and vacuum concentration with Speedvac. Pink line corresponds to refractive index signal while black line is zinc concentration.

Further separations of the low molecular weight Zn fraction by high performance liquid chromatography (HPLC) were performed subsequently, using first a hydrophilic interaction liquid chromatography (HILIC) amino column (Fig. 4a), followed by fractionation on a Superdex 10/300 gel filtration peptide column (SEC) (Fig. 4b), with a step of vacuum concentration in between. The fraction resulting from the SEC step was then used for further characterization.

### Characterization and mass spectrometry of a purified LMW Zn complex

Overall, the chromatographic behaviour of the LMW Zn fraction suggests that it consists of one single compound responsible for zinc binding and accumulation. Its elution was not accompanied by UV absorption which would be expected for metal thiolates like those present in MTs or phytochelatins (PCs). Analysis by ICP-OES confirmed the absence of any sulphur. The occasional detection of phosphorus, on the other hand, was not consistent and was probably due to the presence of impurities.

Overall, the chromatographic methods did not allow classifying the Zn binding compound more precisely. In order to further characterize the still unknown compound, we then applied methods of a mass spectrometry. Although gel chromatography on both, Sephacryl S-100 and Superdex peptide columns suggested an apparent molecular weight of the Zn-containing compound around 2,000 Da, the observed ion masses detected by an electrospray ion source with a differential mobility analyser coupled to an atmospheric-pressure-interface time-of-flight mass spectrometer (ESI-UDMA-APi-TOF-MS) were certainly smaller, with the largest Zn containing mass being 837 Da.

**Figure 5:**
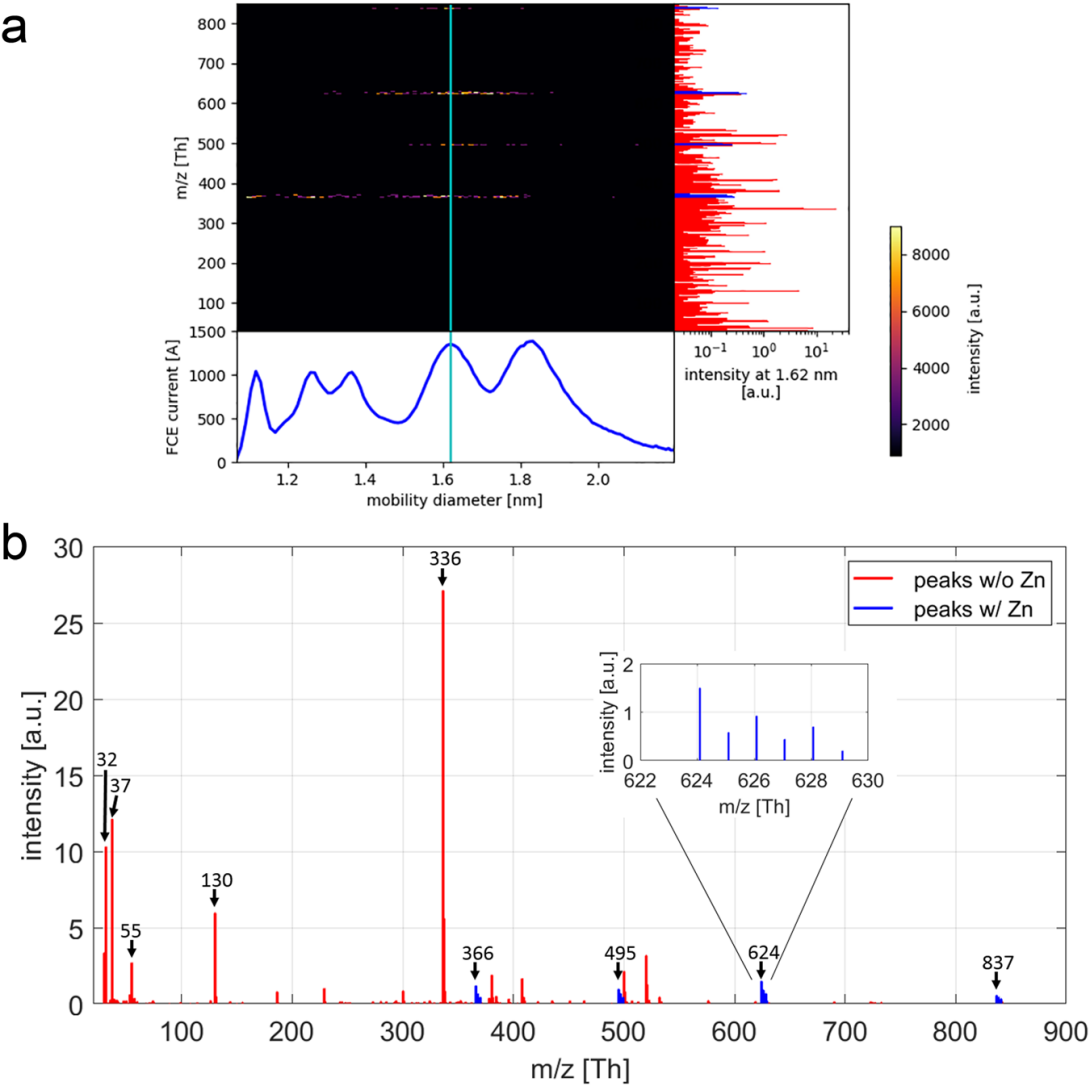
(**a**) 2D-mass-mobility plot of the LMW Zn complex showing ion intensities of Zn containing mass peaks using ESI-UDMA-APi-TOF mass spectrometry. Lower panel, ion mobility diameter recorded with the Faraday cup electrometer (FCE). The cyan line marks the mobility diameter of 1.62 nm. Right panel, mass spectrum recorded at the mobility diameter of 1.62 nm. Zn containing ion peaks are shown in blue. (**b**) Detailed mass spectrum showing four zinc containing peaks with characteristic zinc isotope profile.

A voltage scan with the UDMA of the ESI-generated cations resulted in the ion mobility spectrum reported in the bottom panel of Fig. S3. The mobility spectrum revealed the presence of five distinct peaks at mobility diameters of 1.1, 1.27, 1.36, 1.62 and 1.8 nm. The main panel of Fig. S3 showed a 2D-mass-mobility plot of the intensities for each nominal mass-to-charge ratio (m/z) in the mass range of 30 to 850 Da obtained with the Atmospheric Pressure interface Time Of Flight mass spectrometer (ioniAPi-TOF). Above 850 Da no significant ion peaks were detected. All ion peaks in the mass spectrum are singly charged as all corresponding isotope peaks are separated by one mass unit. Screening the 2-D-mass-mobility plot shown in Fig. S3 for ions containing the element Zn and allowing for other elements such as C, N, O, and H revealed the presence of four corresponding peaks at nominal masses of 366, 495, 624 and 837 Da, respectively. Figure 5a shows the 2D-mass-mobility plot of Zn containing ion peaks together with the mobility diameter. All four peaks show up simultaneously at a mobility diameter of 1.62 nm marked by a cyan line in Fig. 5a. This behaviour of the LMW Zn compound did not fit to any compound of biological origin listed in The National Institute of Standards and Technology (NIST) database.

## Discussion

Terrestrial gastropods are able to accumulate high concentrations of transition metals in their midgut gland^13^. In this organ, metals such as Zn^2+^, Cd^2+^ and Cu^+^ ions can either be bound specifically to MTs and/or low molecular weight ligands such as PCs, unspecifically to high-molecular-weight proteins^11^, or be inactivated by cellular compartmentalization into so-called granules^14–17^. This sequestration into membrane-enclosed granules is wide spread in mollusks leading to inorganic precipitates with phosphates^17^.

### The role of metallothioneins

In pulmonate snails, MTs serve as principal cellular buffers for binding Cd^2+^ and Cu^+^. Tolerance of excess amounts of trace elements in the environment is facilitated by induction of MT genes and is associated with their involvement in metal detoxification. In aquatic invertebrates MTs can be induced by Zn^2+^ and act to store for enzymatic and metabolic metal ion demands^18^. In crustaceans, Zn^2+^ bound to MT is required for the enzyme carbonic anhydrase, however, Zn^2+^ does not induce MT expression^19^. Also Coombs (1974) demonstrated before that MTs were not involved in Zn^2+^ binding in the oyster *Ostrea edulis*. Instead, Zn^2+^ is sequestered using MT-independent metabolic pathways involving complexation to low-molecular weight compounds^20^. LMW Zn complexes were also observed in mussel’s kidney^21^.

Vertebrate MTs can normally form heterometallic complexes (involving Cd^2+^, Zn^2+^, and Cu^+^) simultaneously, although in some vertebrate MTs domains with a distinct preference for a certain metal ion species are encountered^22^. It was hypothesized that during MT synthesis the proteins are initially loaded with zinc ions due to their higher cellular abundance, and that Zn^2+^ is later replaced by other metal ions like Cd^2+^, Hg^2+^, or Ag^+^, thereby detoxifying these harmful metals.

Interestingly, terrestrial gastropod MTs do not display a particular affinity for Zn^2+^, neither under normal physiological conditions, nor when exposed to increased levels of Zn^2+^ or other metal ions. This is also the case for the MT isoforms of *Arion vulgaris*^4^. This apparent lack of affinity of gastropod MTs for Zn^2+^ is a remarkable difference to most vertebrate MTs, which always possess a considerable high binding affinity for Zn^2+^, rendering in most cases mixed metal complexes with a high content of Zn^2+ ^^23^. In contrast, the results of the present study demonstrate that Zn^2+^ ions in the midgut gland of *Arion vulgaris* are consistently associated with low-molecular mass ligands in a molecular weight range of about 2,000 Da (Fig. 3).

In many other mollusc species Zn^2+^ is also associated with LMW compounds. In addition, greater proportions of this metal ion are always detected in insoluble cellular fractions^3,8,11,12,15,24–28^. The present study shows, moreover, that significant proportions of soluble Zn in the midgut gland of *Arion vulgaris* are bound to high-molecular-weight fractions (≥100 kDa), and LMW compounds (Fig. 3). Cytosolic Zn^2+^ bound to LMW complexes (1–4 kDa) was detected repeatedly in the midgut gland of *Helix pomatia*^11,12,14^, *Littorina littorea*^29^ and other molluscan species^20^, but it was never fully characterized so far. By means of histological and histochemical methods, Zn^2+^ in *Arion ater* was detected mainly in lipofuscin granules of excretory cells, as well as in the perinuclear cytoplasm and in calcium spherules of calcium cells, and occasionally also in the cytoplasm and brush border of digestive cells^30^. In the present study, Zn^2+^ was detected after 15 days of metal exposure in the midgut gland of *Arion vulgaris*, and localized exclusively in calcium cells of this organ, where the metal was visible in cytoplasm and, to a minor extent, in calcium granules (Fig. 2). Similar observations were reported for *Arion rufus* exposed to Zn^2+^ for a period of 27 days^27^. Recio *et al*. (1988) explained this variability in Zn distribution by differences in the level of exposure^30^. In *Helix pomatia*, most of the organs accumulate Zn^2+^, but for final storage the majority of the metal (about 70%) is transported to the midgut gland, from which it may be gradually excreted^31^. This corroborates the central role of the midgut gland for metal storage and detoxification in terrestrial snails and slugs.

### The role of phytochelatins (PCs)

Well characterized metal-binding ligands originally observed in plant cells and more recently also reported from invertebrate animals, are PCs^32,33^. These are enzymatically synthesized oligomers of glutathione, consisting of 2 to 6 glutathione units with a molecular weight ranging from 0.5 to 1.5 kDa. They can bind metal ions through the free thiols of their cysteine residues^33^. PCs are synthesized by phytochelatin synthase (PCS), an enzyme which has already been detected in several molluscan species^34^.

The freshwater snail *Biomphalaria glabrata*, for example, synthesizes PCs upon exposure to Cd^2+^ (Supplementary Information Fig. S1). When comparing these data with those of *Arion vulgaris*, it becomes clear that only traces, if any, of PC2 were observed in the midgut gland of the slug, and they are not inducible by metal exposure (Fig. S1). In the present study, the cysteine molar concentration of PC2 is 400 times lower than that of MTs, assuming that all Cd^2+^ seen in Fig. 3b resembles the total concentration of CdMT. The partially purified LMW Zn from *Arion vulgaris* is essentially free of any PC2. These results strongly suggest that PCs are not part of the low-molecular-weight zinc-binding compounds isolated from *Arion vulgaris*.

### Possible zinc chelators

Coombs (1974) described a LMW Zn complex from the oyster, *Ostrea edulis*, that serves as a freely available mobile Zn^2+^ ion pool for metal-dependent enzymatic systems^20^. This was identified as homarine, which was also reported from three species of echinodermata, seven species of arthropoda and eight species of mollusca^35^. Polychronopoulos *et al*.^36^ showed that homarine is a common and abundant metabolite in several marine molluscs^36^. In separations of mollusc extracts (*Ostrea edulis, Littorina littorea*) by SEC chromatography, homarine was shown to co-eluate with a major fraction of Zn^2+ ^^29,37^. In our studies, however, we can exclude the presence of homarine in the low molecular weight Zn fractions from the midgut gland of *Arion vulgaris*. This is shown by comparing the chromatographic behaviour of commercially available homarine on gel filtration chromatography, which differs from that of the LMW Zn compound of *Arion vulgaris*. Furthermore, we could not detect any peak at 137 Da in the mass spectra of partially purified LMW Zn, as would be expected for homarine.

### Characterization of the low molecular weight zinc binding compound (LMW Zn) of Arion vulgaris

**Figure 6:**
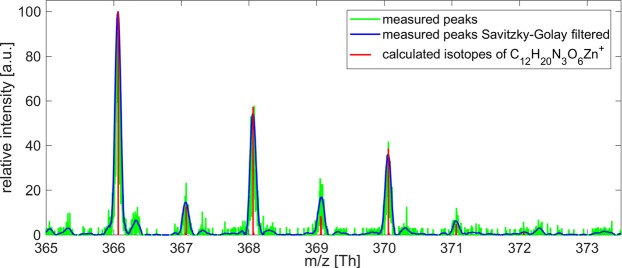
Measured isotopic pattern of the m/z 366 peak recorded as fragment ions at a mobility diameter of 1.62 nm. Also shown is the calculated isotope pattern of C_12_H_20_N_3_O_6_ Zn^+^, which gives an excellent match.

Purification of the Zn containing compounds by chromatography suggested that there is only one LMW Zn compound present (Figs. 3 and 4). The lack of UV absorbance at 260 nm indicates, moreover, that the corresponding fractions are devoid of metal thiolates. Further evidence that no SH groups are involved is given by the attempts to measure PCs. All SH containing compounds should give a fluorescent signal during these analyses. The UDMA scan (Fig. 5a) indicates that the four Zn containing ion peaks observed in the mass spectrum correspond to one LMW Zn complex with a mobility diameter of 1.62 nm and a molecular mass of 837 Da. Ion peaks at 624 (837 minus 213), 495 (624 minus 129) and 366 (495 minus 129) Da (Fig. 5b) are fragment ions from the parent ion found at 837 Da losing neutral fragments of 213 Da and 129 Da, respectively. Similarly, the ion peak at 366 Da occurs also at smaller mobility diameters of 1.1 nm and 1.27 nm, respectively (Fig. 5a) and can be explained by fragmentation of a neutral Zn complex. Figure 6 reports the measured isotopic pattern of the m/z 366 peak recorded as fragment at a mobility diameter of 1.62 nm. Shown is the calculated isotope pattern of C_12_H_20_N_3_O_6_ Zn^+^, which gives an excellent match with nicotianamine (NA). Tsednee *et al*. (2016) analysed with ESI-MS metal-NA complexes^38^. They observed that Zn(II)-NA produces singly charged positive ions with the formula [NA-H + Zn(II)]^+^ corresponding to 366.064 Da in the ESI ion source. Therefore, we speculate that the Zn-containing ion peak recorded at mass 366 has the structure of [NA-H + Zn(II)]^+^ and is the result of fragmentation occurring in the ion source as well as in the mass spectrometer from a LMW Zn complex having a m/z of 837 Da (positive ion mass) corresponding to a mobility diameter of 1.62 nm. While gel chromatography suggested an apparent molecular weight about 2,000 Da, the highest mass detected in ESI-UDMA-MS was below 900. The mass at 366 fits to a NA-Zn complex. Nicotianamine is a phytosiderophore, but the functionality of nicotianamine synthase has so far never been shown in the animal kingdom, although some gene bank entries suggest the presence of a putative *nicotianamine synthase* gene (e.g. *Folsomia candida*, OXA59372.1).

Thus, all our data are in line with a sulphur-free, non-aromatic skeleton of the LMW Zn compound. Notably the absence of sulphur discriminates it from most Cd^2+^-binding compounds. This is corroborated by the arguments of Martin (1986) that sulphur containing compounds have a higher affinity for Cd^2+^ than for Zn^2+^, while oxygen or nitrogen centred chelators have higher affinities for Zn^2+^ as compared to Cd ions^39^. This simple chemical rule may explain the segregation between Zn-binding compartments and Cd/Cu-selective compounds in *Arion vulgaris*.

## Conclusions

One of the most important findings of this paper is the fact that in *Arion vulgaris* the pathway of Zn^2+^ ions is clearly separated from the pathway used for Cd^2+^ ions, which is specifically associated with Cd-selective metallothioneins. Although there is no unifying metal-specific role for metallothioneins across animal phyla, it is speculated that metal-discriminating pathways are positively correlated with metal selectivity of the respective metallothionein systems.

## Material and Methods

### Chemicals

Unless otherwise stated, all reagents and solvents were purchased from Carl Roth GmbH (Karlsruhe, Baden-Württemberg, Germany), and were of analytical or HPLC grade.

### Animals

Specimens of *Arion vulgaris* were collected in Innsbruck, Austria, in June-August 2015. The animals were kept in groups of 25 individuals each in plastic boxes (18 × 27 × 11 cm) on moistened garden soil at constant conditions (18 °C, 12 hours light/dark rhythm, 80% humidity). During an acclimatization period of 3 weeks, they were fed on clean lettuce (*Lactuca sativa*) four times per week. Metal-treated slugs were fed up to 15 days on metal-enriched lettuce daily. Metal contamination of the diet was achieved by soaking lettuce leaves in a Zn solution (ZnCl_2_) of 50 mg · L^−1^ or Cd solution (CdCl_2_) of 1 mg · L^−1^ for one hour. According to Austrian legislation no license is required for research on invertebrates like slugs and snails.

### Metal analysis

Tissue aliquots (6 specimens) were dried in U25 oven (Memmert, Schwabach, Germany) at 60 °C for several days until constant weight. Samples (1–20 mg dry weight) were digested with 1 mL of a mixture of 36% nitric acid (Suprapur®, Merck, Darmstadt, Germany) and distilled water (1:1) in 2 mL polypropylene safe-seal microtubes (Sarstedt, Nümbrecht, Germany) on a heated aluminum block at 70 °C until the remaining solution was clear. After dilution with distilled water to a total volume of 2 mL, metal analysis were carried out by flame atomic absorption spectrophotometer (AAS Perkin-Elmer, model 2380, Waltham, USA; for details see Dallinger *et al*., 1989^40^).

### Histochemistry

Midgut gland aliquots were embedded in Tissue tek® (Sakura, Torrance, USA), frozen in isopentane, liquid nitrogen and stored at −80 °C. Frozen samples were sectioned (10 µm thick) on a cryostat (−20 °C), thaw-mounted on glass slides Dako REALTM (Dako, Glostrup, Denmark), dried overnight, and stored at −80 °C. For histochemical detection of Zn, sections were stained with 0.01% dithizone in an acetone-water solution for 5 min^41^. For fluorescent detection of Zn, sections were stained at 4 °C with 100 µL of 0.01% TSQ in acetone for 1 min and then air-dried^42,43^.

### Chromatographic separations

Dissected midgut gland of slugs (samples about 3 mL, n = 3) and buffer, containing 25 mM Tris(hydroxymethyl)aminomethane hydrochloride (Tris-HCl) pH = 7.5, 5 mM ß-mercaptoethanol, and 0.1 mM phenylmethanesulfonyl fluoride (PMSF) were mixed (1:3) and homogenized. After centrifugation (15,000 rpm, 4 °C, 15 min), supernatant was filtered (0.2 µm) and applied to gel chromatography column (Sephacryl^TM^ S-100 High Resolution, 310 × 20 mm, GE Healthcare, Little Chalfont, UK) pre-equilibrated with 25 mM Tris-HCl pH = 7.5, and 5 mM ß-mercaptoethanol. Column was eluted with same buffer at a flow rate of 2 mL · min^−1^ and fractions were collected every 2 min. UV-absorption at 254 nm was recorded (U-2000 UV spectrophotometer, Hitachi, Tokyo, Japan). Zinc and cadmium concentrations were measured in all fractions by flame atomic absorption (AAS Perkin-Elmer, model 2380).

Zinc containing low molecular weight fractions were pooled and concentrated on a SpeedVac Savant SC110 centrifuge (Thermo Fisher Scientific, Waltham, CA, USA). High performance liquid chromatography were performed on an LC 10-AD liquid chromatograph (Shimadzu, Kyoto, Japan) with a LiChrospher NH_2_ HPLC column (5 μm particle size, L × I.D. 15 cm × 4.6 mm) (Merck). Buffer A consisted of 95% acetonitrile in 10 mM NH_4_HCO_3_ and buffer B consisted of 5% acetonitrile in 10 mM NH_4_HCO_3_. Fractions were eluted with a two-step, linear gradient consisting of 5–20% B for 0–20 min followed by 20–50% B from 20–30 min. The column was maintained at ambient temperature and run at a flow-rate of 0.5 mL · min^−1^.

After the previous separation, zinc-containing fractions, pooled and concentrated on a SpeedVac were fractionated by HPLC with a Superdex peptide 30/100 GL column (GE Healthcare) with 10 mM NH_2_HCO_3_ buffer or water.

### Mass spectrometry

Mass spectrometry was done by combining an electrospray ion source with a differential mobility analyser coupled to an atmospheric-pressure-interface time-of-flight mass spectrometer (ESI-UDMA-APi-TOF-MS^44^) shown in Fig. S2. Purified LMW Zn complex in distilled water was introduced through a capillary into the electrospray ion source generating positively as well as negatively charged ions. The size-distribution of the ions was characterized with a differential mobility analyser. The ions could be detected either through a Faraday cup electrometer or a mass spectrometer.

### Statistical analysis

Since most values for tissue metal concentrations failed to pass the Shapiro–Wilk normality test and the equal variance test, non-parametric statistical methods were applied as mentioned below. A two-way analysis of variance (ANOVA) was performed in Sigmaplot 12.5 (SYSTAT software, San Jose, CA, USA) applying the Holm–Sidak method for pairwise and multiple comparisons, with a significance level of p ≤ 0.05. A home-made graphics program (RLplot 1.5.6a) (https://www.uibk.ac.at/zoology/download/rlsoft/index.html.en) was applied to design the plots which were finally edited with Adobe Illustrator CC (Adobe Inc., San Jose, California, United States).

## Supplementary information

Supplementary information

## Data Availability

The raw data for this work can be downloaded from: https://www.uibk.ac.at/zoology/download/srep-19-07962-t/.
