# Supplementary material for: Distinct pathways for zinc metabolism in the terrestrial slug Arion vulgaris
Source: Sci Rep. 2019 Dec 27;9:20089. doi: 10.1038/s41598-019-56577-7 (PMC6934671; doi:10.1038/s41598-019-56577-7)
Supplement: Supplementary file 1 — Supplementary information [file 41598_2019_56577_MOESM1_ESM.pdf]

Distinct pathways for zinc metabolism in the terrestrial slug, *Arion vulgaris*

Supplementary Information

Martin Dvorak[1)], Raimund Schnegg[1)], Willy Salvenmoser[1)], Oscar Palacios[2)], Herbert Lindner[3)], Oliver Zerbe[4)], Armin Hansel[5)], Markus Leiminger[5)], Gerhard Steiner[5, 6)], Reinhard Dallinger[1)*], Reinhard Lackner[1)*]

1) Institute of Zoology and Center of Molecular Biosciences, University of Innsbruck, Technikerstrasse 25, A-6020 Innsbruck, Austria

2) Departament de Química, Facultat de Ciències, Universitat Autònoma de Barcelona, E-08193 Cerdanyola del Vallès, Barcelona, Spain

3) Innsbruck Medical University, Biocenter, Division of Clinical Biochemistry, Innrain 80 A-6020 Innsbruck, Austria

4) Department of Chemistry, University of Zurich, 8057 Zurich, Switzerland

5) Institute for Ion Physics and Applied Physics, University of Innsbruck, Technikerstrasse 25, A-6020 Innsbruck, Austria

6) GRIMM Aerosol Technik Ainring GmbH & Co. KG, 83404 Ainring, Germany

* Corresponding authors:

reinhard.dallinger@uibk.ac.at

reinhard.lackner@uibk.ac.at

Phytochelatins in Gastropods

Determination of phytochelatins was based on a modified HPLC protocol with detection of fluorescent derivatives described by Kawakami et al. (2006). Briefly, homogenized midgut gland in 0.1M TFA, 10 mM EDTA and 10 mM TCEP (1:3 w/v) was centrifuged at 13,200 rpm for 20 min at 4 °C. 50 µl of supernatant was pre-derivatized with 150 µl of 0.1 M NH_4HCO_3 and 50 µl 0.1 M TCEP in 0.1 M NH_4HCO_3 for 1 hour at room temperature. The disulfide bonds were converted to sulfhydryls by adding 615 µl HEPES buffer and 25 µl 20 mM TCEP and incubated for 10 min at 45 °C, followed by adding 10 µl of 250 mM monobromobimane and derivatized for 45 min at 45 °C in the dark. After termination of derivatization by addition of 100 µl 1 M TFA, the sample was centrifuged for 1 min at 13,200 rpm. 100 µl of the sample were then applied to reverse phased HPLC using a LiChrospher 100 RP-18 (5 µm) column (Merck, Darmstadt, Germany) maintained at 40 °C. The thiol-bimane derivates were eluted in a linear gradient of buffer A consisting of 0.1% TFA and buffer B consisting of 0.1% TFA in acetonitrile (0-5% B for 0-5 min, 5-25% B for 5-30 min and 25-100% for 30-40 min) at a flow rate 1 ml · min^{-1}. Fluorimetric detection was achieved with 380 nm (excitation) and 470 nm (emission) wavelengths. A calibration curve was obtained with 25 µg of phytochelatin standards (PC2-PC6) derivatized in the same way as the samples. Fractions were manually collected and provided for further MS analysis to confirm individual oligomers of phytochelatins. These data show that phytochelatins do not play a significant role in *Arion vulgaris*. The inducibility of phytochelatin synthesis appears to be species-dependent. In contrast to *Arion vulgaris* phytochelatins are inducible in *Biomphalaria glabrata*.

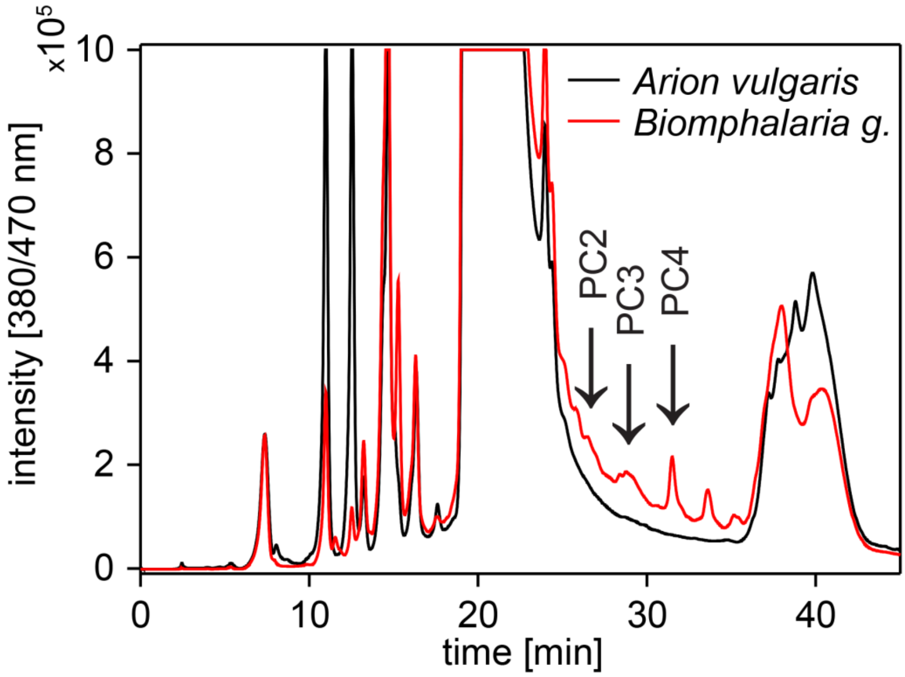

Figure S1: Determination of phytochelatins by HPLC with fluorescent detection of derivatives of monobromobimane. Comparison of Cd exposed *Arion vulgaris* (black line) and *Biomphalaria glabrata* (red line) midgut gland. PC2, PC3, and PC4 show the position of different oligomers of phytochelatins.

Mass spectrometry

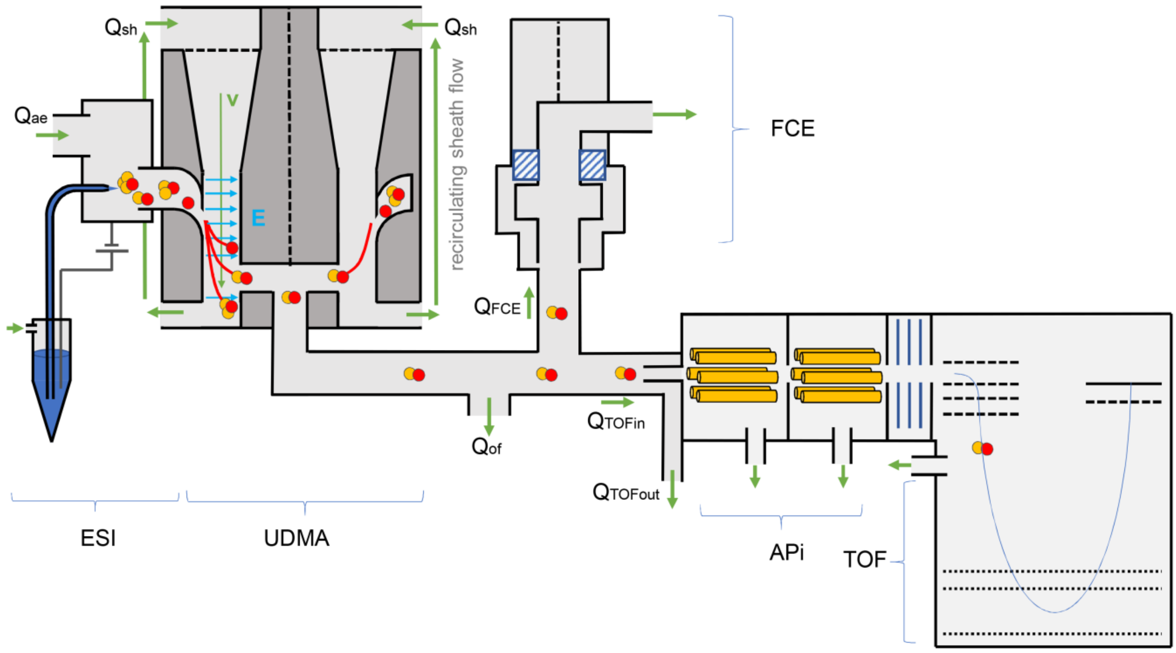

Figure S2: Purified LMW Zn complex in distilled water was introduced through a capillary into the electrospray ion source (ESI) generating positively as well as negatively charged ions. These ions were introduced into a laminar flow of clean and dry (RH < 2%) air Q_{ae} (14 standard liter·min^{-1}) transporting the ions to a differential mobility analyzer (UDMA[2]). The ions could be detected either through a Faraday cup electrometer (FCE[3]) measuring ion current or an Atmospheric Pressure interface Time Of Flight mass spectrometer (ioniAPi-TOF) from Ionicon Analytk GmbH, Innsbruck Austria[4]. The ioniAPi-TOF has a mass resolution (m/Δm) of ~6000. We used the following flow settings: Q_{FCE} and Q_{TOFin} each 6 SL/min, Q_{TOFout} 5 SL/min, and an overflow Q_{of} of typically 2 SL/min.

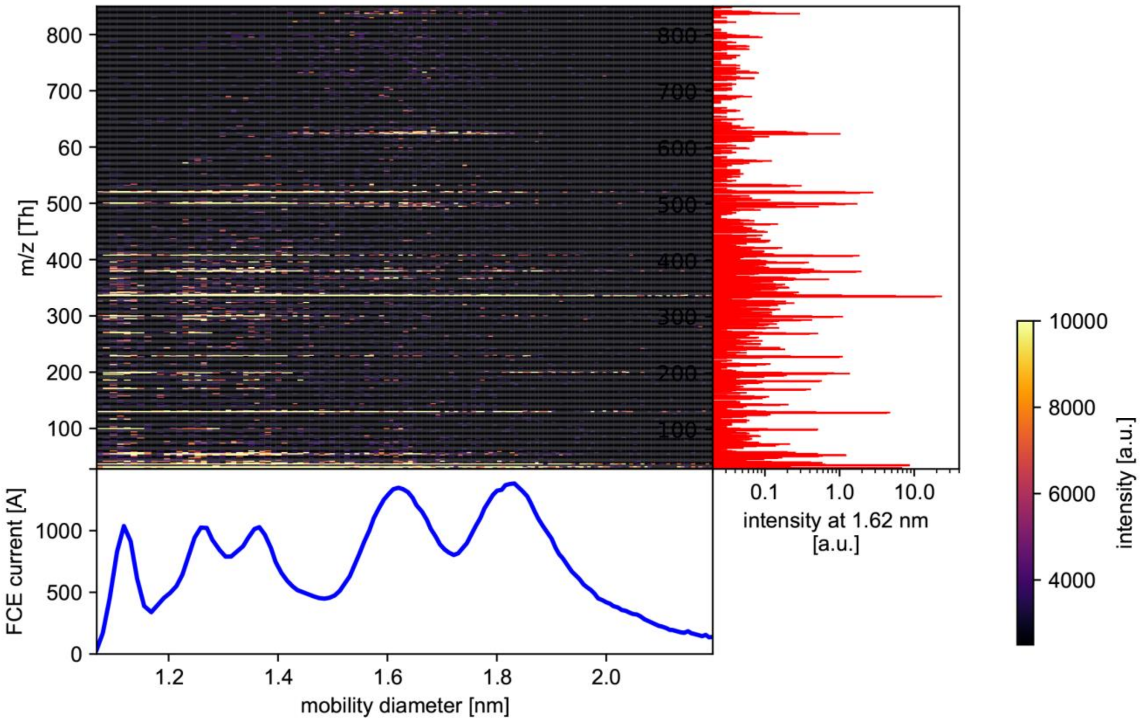

Figure S3: The ion current of the Faraday cup electrometer (FCE) revealed five peaks while scanning the voltage of the UDMA (lower panel). These peaks correspond to mobility diameters of 1.1, 1.27, 1.36, 1.62 and 1.8 nm, respectively. The mobility diameter was determined in a few steps. First, the UDMA voltage was converted to units of ion mobility using the relationship $Z_i = Z_{Ref} \cdot V_{Ref}/V_i$ [2] and the mobility 0.97 cm²/Vs of the reference ion, the charged monomer of Tetra-Heptyl-Ammonium Bromide[5]. Then, the mobility diameter D_p after Milikan-Fuchs was determined [6] via $Z_p = 2.2458 \cdot 10^{-22} \cdot D_p^{-1.9956}$.

The main panel shows a 2D-mass-mobility plot of the intensities of each nominal mass-to-charge ratio (m/z) in the mass range of 30 to 850 Da obtained with the ioniAPi-TOF. Above 850 Da no significant ion peaks were detected. The intensities are corrected for the mass dependent transmission efficiency of the mass spectrometer as described in Leiminger et al. (2019).

A mass spectrum integrated over 10 min at the mobility diameter of 1.62 nm is shown in the panel at the right.

References list

1. Kawakami, S. K., Gledhill, M. & Achterberg, E. P. Determination of phytochelatins and glutathione in phytoplankton from natural waters using HPLC with fluorescence detection. *Trends Anal. Chem.* **25**, 133–142 (2006).

2. Steiner, G., Attoui, M., Wimmer, D. & Reischl, G. P. A Medium Flow , High-Resolution Vienna DMA Running in Recirculating Mode. *Aerosol Sci. Technol.* **44**, 308–315 (2010).

3. Winklmayr, W., Reischl, G. P., Lindner, A. O. & Berner, A. A new electromobility spectrometer for the measurement of aerosol size distributions in the size range from 1 to 1000 nm. *J. Aerosol Sci.* **22**, 289–296 (1991).

4. Leiminger, M. *et al.* Characterisation of the transfer of cluster ions through an atmospheric pressure interface time-of-flight mass spectrometer with hexapole ion guides. *Atmos. Meas. Tech.* **12**, 5231–5246 (2019).

5. Ude, S. & Mora, J. F. De. Molecular monodisperse mobility and mass standards from electrosprays of tetra-alkyl ammonium halides. *J. Aerosol Sci.* **36**, 1224–1237 (2005).

6. Mäkelä, J. M., Riihelä, M., Ukkonen, A., Jokinen, V. & Keskinen, J. Comparison of mobility equivalent diameter with Kelvin-Thomson diameter using ion mobility data. *J. Chem. Phys.* **105**, 1562–1571 (1996).
